# Calling for the next WHO Global Health Initiative: the use of disruptive innovation to meet the health care needs of displaced populations

**DOI:** 10.7189/jogh.08.010303

**Published:** 2018-06

**Authors:** Robert MT Staruch, Anair Beverly, Jason K Sarfo-Annin, Sian Rowbotham

**Affiliations:** 1 John A. Paulson School of Engineering & Applied Sciences, Harvard University, Cambridge, Massachusetts, USA; 2 Department of Anaesthesiology, Brigham & Women’s Hospital, Boston, Massachusetts, USA; 3 Fulbright Scholar & Master’s in Public Health Candidate, T. H. Chan School of Public Health, Harvard University, Cambridge, Massachusetts, USA; 4 Finn Church Aid, Athens, Greece; * Joint first authorship

In the last 10 years, political unrest, religious disputes, genocide and civil war have caused the greatest displacement of people on record [1,2]. In 2015 the United Nations Refugee Agency (UNHCR) recorded 65.3 million displaced people globally, of whom over half are children and adolescents [3]. 6% of these displaced people are currently hosted in Europe, whilst 40% are in Middle East and North Africa [2]. Turkey alone hosts 2.6 million refugees and Pakistan another 1.5 million. As half of all refugees globally come from three countries, Syria, Afghanistan and Somalia. With each of these experiencing ongoing conflict, there is no clear timeframe for resolution, and with a poorly co-ordinated international response the challenge of managing health in these refugees will go from acute to chronic [3-5].

The World Health Organization (WHO) is the arm of the United Nations concerned with the delivery of public health. Their 2014-15 budget totaled US$ 4 billion. In collaboration with the World Bank, and other partners, the WHO is responsible for administering the International Health Partnership (IHF). The IHF is a partner organization linking governments, development agencies and other groups with a common goal to improve health care in developing nations. The World Health Organization therefore occupies a key financial and organizational position, and this role demands both proactive and reactive strategies in the mission to improve health care across both settled and displaced populations. The WHO’s Global Health Initiatives serve to strengthen the health systems in developing nations by delivering additional funds for specific health care interventions; such as immunization programs or fighting infectious diseases such as Malaria or Tuberculosis. Recent WHO Global Health initiatives have also included research to measure and improve global surgery outcomes [6,7].

“Disruptive innovation” describes technological developments which create new markets and values within an existing market, therefore displacing historic market leaders and products. This phenomenon was described by Harvard Business School Prof. Clayton [8]. M Christiansen in 1997 [8,9] and from its roots in business, has been modelled and described with case studies in both health care and major social change. The concept of Disruptive Innovation is therefore highly pertinent to refugee health care, where traditional economic drivers are absent (**Table 1**).

**Table 1:** Some examples of disruptive innovation [9]*

Disruptor	Disruptee
Personal Computers	Mainframe and mini computers
Mini mills	Integrated steel mills
Cellular phones	Fixed line telephony
Community Universities	Four-year Universities/Fast Track Universities
Discount retailer stores	Full-service department stores/Shopping Malls
Retail medical clinics	Traditional doctor’s offices

*Demonstrations of Low Cost – High Tech disruptive technology emerging into the market. Such devices are currently spearheaded by private biotech companies responding to market forces, rather than the directions of global health communities advocating the needs of a large patient group.

Understanding economic principals behind disruptive innovation is important for those championing provision of low-cost health care products for displaced people with minimal or no disposable resources. However, increased longevity, a growing burden of chronic disease and significant health inequalities make value of health care an increasingly scrutinised metric even in more developed economies. This is true in the USA, one of the largest health care markets in the world, in the UK, and across economically developed nations worldwide [9].

Host nations’ perceived or actual capacity to integrate refugees into existing health care systems has been exceeded [1]. The responsibility of any individual nation or multinational organisation for managing migrant or stateless people’s health remains debated in political and humanitarian arenas. In Europe alone, millions cannot obtain access to their host nation’s private or public health care systems. Barriers include detainment in camps without basic health care services and ineligibility to enrol in publicly funded services from lack of identification paperwork or asylum-seeker status. For displaced people, particularly those gathered in camps such as Calais, France, or Thessaloniki, Greece. Non-Governmental Organisations (NGOs) play a significant role in delivering health care.

**Figure:**
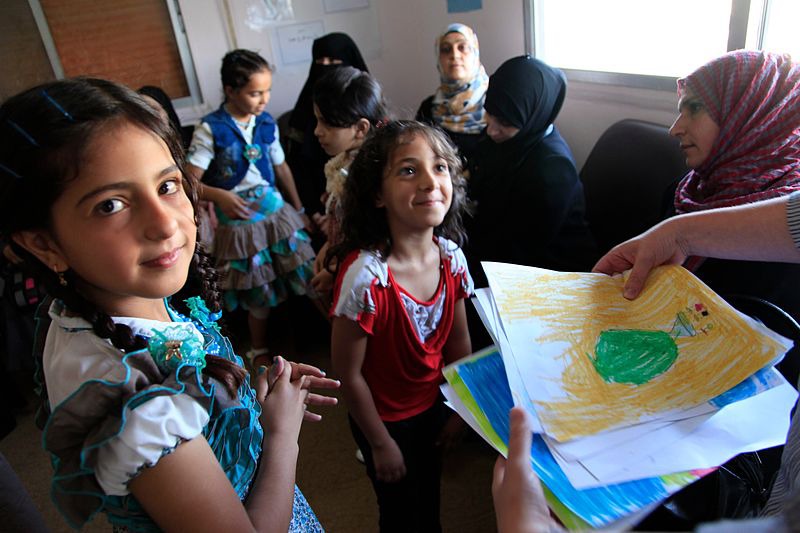
Photo: Refugee children from Syria at a clinic in Ramtha, northern Jordan (Russell Watkins/Department for International Development, UK, via Wikimedia Commons)

Healthcare quality, regardless of setting, is measured by timeliness, efficacy, evidence basis, patient centeredness and cost-effectiveness. Refugees have both similar and unique needs compared to citizens, but very different resources. In the words of philanthropist Bill Gates, “one of the biggest challenges today is to make scientific innovation improve the lives of the poorest”. Redoubled efforts in technological innovation with the explicit target of developing low cost materials, methods and applications for low cost health care delivery are one way the academic community can response to humanitarian crisis.

The specific medical and surgical needs of refugees in camps depends on their demographics, the climate, season, presence of violent armed conflict and endemic or epidemic diseases. NGOs rightly focus on highly cost-effective, low-tech primary health care strategies of sanitation, vaccination, and treatment of infectious diseases. However, other common needs are treatment of fractures, burns, wounds, skin diseases and medications and monitoring for chronic diseases like asthma and diabetes. Tests, treatments, devices and data technologies for use in a refugee camps need to be durable, long-life, easy to store and transport and cheap to produce with robust quality assurance and traceability. A disruptive technological solution therefore needs to look beyond merely delivering low cost high volume replica products, but also in introducing new value and significant health care benefit from such an intervention. Medical records, documentation and data collection in these groups are challenging but vital. Simple cloud based storage systems with international access through mobile phone and telecommunication networks might allow refugee health records to be initiated, given increasing access to GPS active cell phones compared to traditional methods. This could even facilitate cross-country follow up by multiple clinicians. Such a system would give this mobile population the health care ‘structure’ to monitor and manage their chronic disease.

Medical technology companies have significant research and development costs and shareholder responsibilities. The resulting expense of new medical devices and instruments is enabled and encouraged in systems using third party payment by governments, purchasing consortiums or insurance companies. This leaves cost-conscious consumers one-step removed from manufacturers and reduces the need for competitiveness based on price. To remain profitable, commercial strategy includes defending patents and maintaining market shares by periodically releasing similar designs with upgraded functions or features at higher prices. It is traditionally perceived as high risk, unprofitable and financially illogical for established companies to research entirely novel technologies and materials which could achieve adequate function for a fraction of the cost.

RAND, a leading non-profit organisation which works to improve policy decisions through research and analysis, described this sea of change in disruptive innovation within a recent report [10].

This report outlines how prior to 2010 Affordable Care Act, health care had been ring fenced from the threat of disruptive innovation. In the field of quality improvement, budget limitation is a key driver to examine processes and eliminate waste. Equally, emerging lower-budget markets invites genuine technological innovation. Many innovative breakthroughs (case studies) come from small R&D firms with Venture Capital Backing (**Table 2**). It is unclear what impact systematic funding via initiatives lead by the WHO, or grants from governments and universities would have. However in the case studies outlined, addressing the needs at the lowest price point in the market has been an opportunity rather than a limitation.

**Table 2:** Case studies

Product	Innovation	Developers	Reference
Thermostable vaccine preparations	Proprietary formulation for biologics and vaccines to allow stability, potency and patency in austere enviroments.	Biotech start-up, MA, USA, Now collaborating with GSK, Sanofi Pasteur, Glaxosmithkline, VBI Vaccines	[11]
Tablet Computers	Low cost tablet computer “Aakash”	Datawind & Government subsidy; Retail cost US$ 35	
Fetal Heart rate monitor	Use of microphones to detect fetal heart sounds for low cost fetal heart rate monitors for mobile phone technology.	Siemens	[12,13]
Drones	Vaccine and blood product delivery to rural Rwanda	UPS & GAVI – US$ 800 000 Grant for R&D	[14]
Onchocerciasis (River Blindness) Test Kit	Onchocerciasis rapid test to help treatment and elimination efforts	PATH – National Institute for Health – National Institute of Allergy and Infectious disease, South Korean scientists. SD. BIOLINE	[15]
AINA	Platform for diagnostic testing of chronic disease using mobile phone technology	JANA Care	[16]
LIMO	Low cost Limb Immobilisation splint	Stanford-India Biodesign collaboration – cost US$ 5	[17]

Efforts like the Public Health Aspects of Migration in Europe (PHAME) [10] and the WHO European health policy framework [6] aim to address some of the issues encountered with unprecedented migration into Europe. However these initiatives don’t describe disruptive technology as a mechanism to improve refugee health care. Clear top down leadership in this field may stimulate a spike in innovation and growth. Systematically bringing together investment and expertise to reverse engineer products from “high-tech” into “low-cost” should be considered a matter of humanitarian urgency. Breaking this cycle of manufacturing unaffordable products requires a departure from traditional business models. The WHO sits well placed to lead a Global Initiative in disruptive innovation for refugee health care improvement. Such a step may tackle the already challenging landscape of effective, tailored health care provision for this population, and stimulate current market leaders to re-evaluate their approaches. Collaboration between the WHO’s Global Health Initiative, targeted grants for academic researchers, and engagement with start-up companies free from shareholder responsibilities could nurture and champion frugal innovation.
